# CVIWM: A Tightly Coupled State Estimation Method for Poultry House Inspection Robots in Structurally Degraded Environments

**DOI:** 10.3390/ani16121780

**Published:** 2026-06-09

**Authors:** Hongfeng Deng, Canhuan Lu, Jiacheng Jiang, Cheng Fang, Tiemin Zhang

**Affiliations:** 1 College of Engineering, South China Agricultural University, Guangzhou 510642, China; 2 State Key Laboratory of Swine and Poultry Breeding Industry, South China Agricultural University, Guangzhou 510642, China; 3 National Engineering Research Center for Breeding Swine Industry, Guangzhou 510642, China

**Keywords:** poultry house inspection robot, state estimation, multi-sensor fusion, tight coupling, AprilTag

## Abstract

Accurate positioning is essential for inspection robots in caged chicken houses, where long straight corridors, sparse textures, and repetitive structures challenge conventional methods. This paper proposes CVIWM (Coupled Visual-Inertial-Wheel Odometry with Markers), a tightly coupled state estimation method that fuses visual, inertial measurement unit (IMU), wheel odometry (WO), and fiducial marker observations within a factor graph optimization framework. Wheel odometry preintegration suppresses IMU horizontal drift and provides absolute scale, while sparse AprilTag markers (10 m spacing) periodically reset accumulated errors. Experiments in an 80 m corridor of a commercial caged chicken house at 0.116 m/s and 0.232 m/s showed that CVIWM achieves average positioning errors of 2.402 cm and 3.253 cm. This high precision ensured reliable image acquisition (image shift <83 pixels), enabling 95.7% dead hen detection and 98.9% egg detection accuracy. CVIWM offers a low-cost, easy-to-deploy, high-accuracy solution for automated poultry house inspection, supporting smart livestock farming.

## Simple Summary

Caged chicken houses have long, narrow corridors with repetitive features, making it difficult for robots to navigate accurately. In this study, we developed a new positioning method called CVIWM that helps a robot know its exact location in such structurally degraded environments. The method combines data from a camera, a motion sensor, wheel measurements, and small visual tags placed along the corridor. Tested in an 80 m long commercial poultry house, our method achieved positioning errors of less than 3.3 cm. This accuracy allowed the robot to take well-centered cage images, enabling the detection of 95.7% of dead hens and 98.9% of eggs. This low-cost, easy-to-deploy solution offers a practical step toward automated health monitoring and more efficient poultry farming.

## 1. Introduction

China is one of the world’s largest producers and consumers of poultry products, and the intensive, large-scale caged chicken farming model has become a pillar of the livestock industry. Routine inspection is a key link for obtaining information on poultry behavior, health status, and environmental conditions. The use of inspection robots to replace manual labor for automated and regular inspection has become an inevitable trend in the development of smart livestock farming [1,2,3].

### 1.1. Importance of High-Precision Positioning for Poultry Health Monitoring

**Figure 1:**
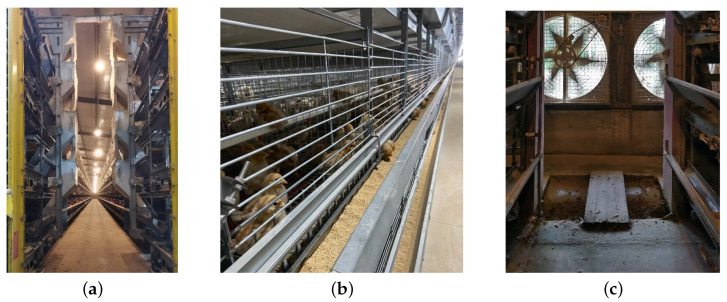
Structurally degraded environment of a caged chicken house. (**a**) Narrow and long straight corridor; (**b**) mesh chicken cage with repeated textures; (**c**) manure pit at the corridor end.

Caged chicken houses exhibit typical structurally degraded characteristics that pose significant challenges for robot positioning. As illustrated in Figure 1, the corridors are long (typically >60 m) and narrow (≈1 m width), flanked by mesh cages that create sparse and repetitive textures. The environment also suffers from uneven lighting, ground stains, and a semi-enclosed structure with a manure pit at the corridor end. These factors collectively degrade the performance of conventional positioning methods: visual odometry (VO) suffers from feature-matching ambiguity, inertial navigation suffers from unbounded drift, and light detection and ranging (LiDAR)-based methods face point cloud sparsity.

High-precision positioning is not only a prerequisite for autonomous robot navigation but also a critical factor for effective poultry health monitoring. Tasks such as dead hen detection rely heavily on the spatial consistency of captured images. When a robot is equipped with a camera to monitor caged hens, even small positioning errors can lead to image misalignment, causing the target cage to shift out of the effective field of view. This would seriously compromise the accuracy and reliability of subsequent health assessments. Therefore, developing a positioning method that is robust to structurally degraded environments is essential for ensuring that inspection robots can provide consistent and accurate spatial information for poultry monitoring.

### 1.2. Related Work

According to different measurement principles, robot positioning technologies can be classified into absolute positioning, relative positioning, and multi-sensor fusion positioning.

Absolute positioning technologies obtain global coordinates by recognizing pre-deployed fiducials. Fiducial-based positioning is widely used in agricultural robotics, mainly including radio frequency identification (RFID) tags, quick response (QR) codes, and AprilTags [4]. Feng et al. [5] used pre-laid magnetic markers and RFID tags for disinfection robots in livestock houses. Moving towards higher precision, Guan et al. [6] integrated AprilTags into a tightly coupled LiDAR inertial odometry via a smoothing and mapping (LIO-SAM) framework as global positioning beacons, achieving average positioning errors of 4.1–7.0 cm (depending on marker density) and lateral navigation deviations below 5.3 cm at speeds of 0.2–0.4 m/s in greenhouse and orchard environments. However, this LiDAR-based approach is costly compared to vision-based solutions. In the vision-based category, Zhang et al. [7] used fiducial markers and factor graph optimization in greenhouse environments, achieving an average error of 5.6 cm. Despite their demonstrated effectiveness, these vision-based marker methods rely on precise deployment and long-term integrity of fiducials, which is difficult to maintain in narrow, equipment-dense caged chicken houses.

Environment-based absolute positioning includes ultra-wide band (UWB) and LiDAR. Niu et al. [8] proposed a deep learning-based ranging error mitigation method for UWB localization systems in greenhouses, using channel impulse response (CIR) as input to predict ranging errors. Their model achieved a mean absolute error (MAE) of 0.1004 m and a root mean square error (RMSE) of 0.1714 m for ranging error prediction in complex greenhouse environments. However, UWB signals are susceptible to multipath effects, and their performance may be severely degraded in environments with dense metal structures [9]. LiDAR positioning offers high accuracy but suffers from feature-matching ambiguity in long, repetitive corridors [10].

Relative positioning technologies estimate position changes by integrating motion sensors. Inertial navigation offers high short-term accuracy but suffers from unbounded cumulative error over long distances [11]. Wheel odometry provides drift-free planar displacement but is affected by scale errors due to slippage [12]. Visual odometry performs well in texture-rich environments but degrades sharply in weak-texture, repetitive environments [13].

In parallel to geometric approaches, learning-based methods have gained traction. Some works use convolutional neural networks (CNNs) to estimate camera pose directly from a single image [14,15]. Others employ recurrent neural networks (RNNs) to model sequential sensor data for end-to-end visual-inertial odometry (VIO) [16,17]. While these methods show promise in rich, textured environments, their performance in texture-less, repetitive, and structurally degraded environments (e.g., long corridors in caged chicken houses) remains a significant challenge [18,19,20]. These learning-based approaches often require massive, diverse datasets for training and may lack the generalization capability and physical constraints (e.g., scale consistency) that traditional geometric fusion provides. Our method, in contrast, relies on a tightly coupled geometric optimization with explicit physical constraints from wheel odometry and markers, ensuring robustness in such challenging, domain-specific scenarios.

Multi-sensor fusion has become a necessary pathway to achieve robust, high-precision positioning. Loosely coupled methods process observations independently [21], while tightly coupled methods jointly optimize raw measurements within a single framework [22,23]. Tightly coupled visual-inertial odometry (VIO), such as visual-inertial navigation system (VINS)-Mono [24], has demonstrated robust performance in texture-rich environments. To further enhance localization accuracy in challenging scenes, researchers have incorporated additional sensor modalities. For instance, a visual-marker-inertial fusion system using sliding window optimization integrates AprilTag observations to provide absolute pose corrections [25], effectively reducing drift in weakly textured environments. However, this approach still relies solely on visual-inertial-marker fusion and lacks direct motion constraints (e.g., wheel odometry), making it susceptible to residual drift between markers, especially in long straight corridors.

### 1.3. Research Gap and Proposed Method

In summary, single positioning technologies face severe challenges in caged chicken house scenarios. Absolute positioning methods are constrained by deployment costs and electromagnetic interference. Environment-based absolute methods (LiDAR) are prone to degradation in long, repetitive corridors. Relative methods (inertial navigation) suffer from unbounded cumulative error. Multi-sensor fusion methods lack effective solutions for structurally degraded environments.

To address these problems, this paper proposes a tightly coupled state estimation method that integrates visual, IMU, wheel odometry, and marker observations within a factor graph optimization framework. The method introduces two key constraints: (1) wheel odometry preintegration provides drift-free planar motion constraints, suppressing IMU horizontal drift and providing absolute scale for monocular vision; (2) sparse fiducial markers serve as absolute pose anchors to periodically reset accumulated errors. All observations are tightly fused within a factor graph optimization framework.

The remainder of this paper is organized as follows: Section 2 describes the materials and methods. Section 3 presents the experimental results. Section 4 discusses the findings. Section 5 concludes the paper.

## 2. Materials and Methods

### 2.1. System Overview

**Figure 2:**
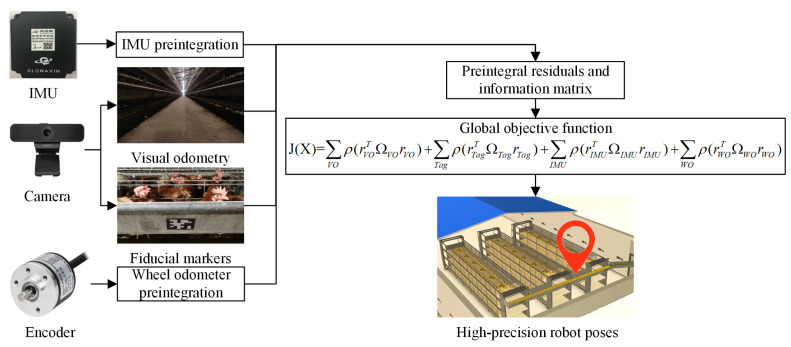
Schematic diagram of the multi-sensor tightly coupled positioning framework of CVIWM.

This paper proposes a tightly coupled state estimation method named CVIWM (Coupled Visual-Inertial-Wheel Odometry with Markers). The overall framework is illustrated in Figure 2. The system integrates four types of sensor observations: (1) visual feature points for relative motion estimation; (2) fiducial markers (AprilTags) for absolute pose constraints; (3) IMU preintegration for high-frequency motion prediction; (4) wheel odometry preintegration for drift-free planar displacement. All observations are jointly incorporated into a tightly coupled global objective function, and the robot’s global pose is computed in real time through nonlinear optimization.

### 2.2. Visual Observation Model

**Figure 3:**
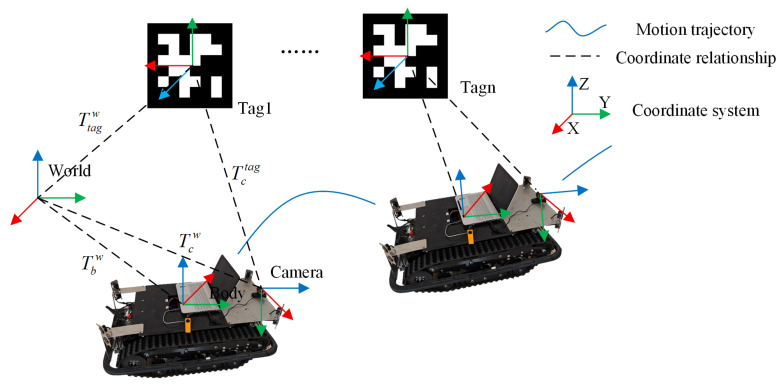
The coordinate relationship in the positioning system.

Visual observations provide two complementary sources of constraints: natural feature tracking for relative motion estimation between consecutive frames (visual odometry), and AprilTag fiducial markers for absolute pose corrections in the global coordinate system. The coordinate relationship in the positioning system is shown in Figure 3.

#### 2.2.1. Camera Projection Model

According to the pinhole camera model, a 3D point with world coordinates $P_{w}={\left[X_{w},Y_{w},Z_{w}\right]}^{T}$ is transformed to the camera frame as:

(1) $$P_{c}=R_{w}^{c}P_{w}+t_{w}^{c}$$

where $P_{c}={\left[X_{c},Y_{c},Z_{c}\right]}^{T}$ represents the coordinates of the point in the camera coordinate system (with $Z_{c}$ being the depth), and $\left[R_{w}^{c}\;|t_{w}^{c}\right]$ is the extrinsic parameter matrix that transforms points from the world frame to the camera frame.

The transformation from the camera frame to the image plane is then given by:

(2) $$p=\pi \left(P_{c}\right)=\frac{1}{Z_{c}}KP_{c}$$

where π(·) is the projection function, $p={\left[u,v\right]}^{T}$ are the pixel coordinates, and *K* is the intrinsic matrix:

(3) $$K=\left[\begin{array}{ccc} f_{x} & 0 & c_{x}\\ 0 & f_{y} & c_{y}\\ 0 & 0 & 1 \end{array}\right]$$

The parameters $f_{x}$, $f_{y}$ are the focal lengths, and $c_{x}$, $c_{y}$ are the principal point coordinates, all of which are obtained through checkerboard calibration.

#### 2.2.2. Visual Odometry from Natural Features

For consecutive keyframes *i* and *j* with matched natural features, the relative camera motion can be recovered by solving epipolar geometry constraints. Let the relative rotation and translation be $R_{ij}^{c}$ and $t_{ij}^{c}$. The reprojection relation is:

(4) $${\hat{p}}_{j}=\pi \left(R_{ij}^{c}\left(\pi^{-1}\left(p_{i},d_{i}\right)\right)+t_{ij}^{c}\right)$$

where ${\hat{p}}_{j}$ is the predicted pixel coordinates in frame *j*, $p_{i}$ is the measured pixel coordinates in frame *i*, $d_{i}$ is the inverse depth of the feature in frame *i*, and $\pi^{-1}$ back-projects a pixel to a normalized ray. By solving the essential or fundamental matrix, $R_{ij}^{c}$ and $t_{ij}^{c}$ are obtained, forming the relative motion measurement $z_{ij}^{VO}=\left(R_{ij}^{c},t_{ij}^{c}\right)$ of the visual odometry.

#### 2.2.3. Uncertainty and Reprojection Residual for Visual Reprojection

Visual uncertainty originates from pixel localization errors. Assuming independent Gaussian noise in the *u* and *v* directions with standard deviation $\sigma_{p}=0.5$ pixel [26], the pixel covariance matrix is:

(5) $$\Sigma_{px}=\sigma_{p}^{2}I_{2}$$

where $I_{2}$ denotes the 2×2 identity matrix.

This pixel error propagates to the 3D point in the camera frame via linearization of the projection model. The covariance of $P_{c}$ is:

(6) $$\Sigma_{P_{c}}=Z_{c}^{2}\cdot J\Sigma_{pn}J^{T}$$

where *J* is the Jacobian mapping the 2D error to the 3D point, and $\Sigma_{pn}$ is the covariance in the normalized plane. The Jacobian of the projection function π with respect to $P_{c}$ is [27]:

(7) $$J_{\pi }=\frac{\partial \pi (P_{c})}{\partial P_{c}}=\left[\begin{array}{ccc} f_{x}/Z_{c} & 0 & -f_{x}X_{c}/Z_{c}^{2}\\ 0 & f_{y}/Z_{c} & -f_{y}Y_{c}/Z_{c}^{2} \end{array}\right]$$

The visual reprojection residual is defined as the difference between the observed and reprojected pixel coordinates:

(8) $$r_{VO}\left(z_{ij}^{VO},X\right)=p_{j}-\pi \left(R_{ij}^{c}\left(\pi^{-1}\left(p_{i},d_{i}\right)\right)+t_{ij}^{c}\right)$$

where X represents the state vector to be optimized.

The covariance matrix of the visual reprojection residual $r_{VO}$ is obtained by error propagation [28]:

(9) $$\Sigma_{ij}^{VO}=\Sigma_{px}+J_{proj}\Sigma_{P_{c}}J_{proj}^{T}$$

where $J_{proj}$ is the Jacobian of the reprojection residual with respect to the 3D point $P_{c}$ (equivalent to $J_{\pi }$).

The information matrix used in optimization is the inverse of the covariance:

(10) $$\Omega_{VO}={\left(\Sigma_{ij}^{VO}\right)}^{-1}$$

#### 2.2.4. Fiducial Marker Definition and Detection

Each AprilTag defines four corners in its own coordinate frame $\left\{tag\right\}$. Let the tag side length be *L*, with the origin at the tag center and the XY-plane coincident with the tag plane. The 3D coordinates of the four corners in $\left\{tag\right\}$ are:

(11) $$Q_{h}^{tag}\in {\left\{\pm \frac{L}{2},\pm \frac{L}{2},0\right\}}_{h=1}^{4}$$

where *h* denotes the index of the *h*-th tag corner.

When the camera observes a tag with known pose, the detection algorithm extracts the pixel coordinates ${\left\{p_{h}\right\}}_{h=1}^{4}$ of the four corners. Since all corners lie on the $Z^{tag}=0$ plane, the camera-to-tag pose $\left[R_{\mathrm{tag}}^{c}|t_{\mathrm{tag}}^{c}\right]$ is obtained by solving a perspective-n-point (PnP) problem.

We employ the efficient perspective-n-point (EPnP) algorithm, which uses four virtual control points to build a linear system via weighted summation. The optimal rigid transformation is recovered by singular value decomposition (SVD).

#### 2.2.5. Global Camera Pose Computation from Fiducial Markers

Given the prior pose of the tag in the world frame $\left[R_{\mathrm{tag}}^{w}|t_{\mathrm{tag}}^{w}\right]$ (obtained by prior surveying), the global camera pose is computed as:

(12) $$\left[R_{c}^{w}|t_{c}^{w}\right]=\left[R_{tag}^{w}|t_{tag}^{w}\right]\cdot {\left[R_{tag}^{c}|t_{tag}^{c}\right]}^{-1}$$

Expanding the above, the camera rotation and translation in the world frame are:

(13) $$\left\{\begin{array}{c} R_{c}^{w}=R_{tag}^{w}{\left(R_{tag}^{c}\right)}^{T}\\ t_{c}^{w}=t_{tag}^{w}-R_{tag}^{w}{\left(R_{tag}^{c}\right)}^{T}t_{tag}^{c} \end{array}\right.$$

where $R_{c}^{w}$ and $t_{c}^{w}$ denote the camera’s rotation and translation (position) in the world frame.

#### 2.2.6. Uncertainty and Reprojection Residual for Fiducial Marker

The uncertainty of AprilTag observations also originates from corner localization errors in the image plane, with standard deviation $\sigma_{tag}=0.3$ pixel. Assuming independent corner localization errors, the joint covariance matrix for all four corners is:

(14) $$\Sigma^{Tag}=\sigma_{tag}^{2}I_{8}$$

where $I_{8}$ denotes the 8×8 identity matrix.

For an AprilTag detected in keyframe *j* with known global pose, the reprojection residual is defined as the stacked vector of projection errors over all four corners:

(15) $$r_{Tag}\left(z_{j}^{Tag},X\right)={\left\{p_{h}^{j}-\pi \left(T_{c_{j}w}T_{wtag}P_{h,tag}\right)\right\}}_{h=1}^{4}$$

where $z_{j}^{Tag}$ represents the AprilTag measurement data in keyframe *j*, $p_{h}^{j}$ are the measured pixel coordinates of the *h*-th corner in keyframe *j*, $P_{h,tag}$ is the homogeneous coordinate of the *h*-th corner in the tag frame $\left\{tag\right\}$, $T_{wtag}$ is the known prior pose of the tag in the world frame, $T_{c_{j}w}$ is the camera pose in the world frame at keyframe *j* (to be optimized).

Due to the high detection accuracy of fiducial markers, the information matrix is assigned a higher weight:

(16) $$\Omega_{Tag}={\left(\Sigma^{Tag}\right)}^{-1}$$

In the optimization framework, the AprilTag factor serves as a strong prior constraint, effectively correcting cumulative drift from visual odometry and inertial navigation, and providing a globally consistent absolute pose reference.

Natural features provide dense relative motion constraints between consecutive frames, while AprilTag markers provide sparse but globally accurate absolute pose corrections. Their complementary characteristics—high-frequency relative constraints versus drift-free absolute anchors—enable robust and accurate state estimation in the optimization framework.

### 2.3. IMU Preintegration

#### 2.3.1. IMU Measurement Model and Kinematics

IMU provides high-frequency measurements of angular velocity and linear acceleration. Let ${\tilde{\omega }}_{k}^{IMU}$ and ${\tilde{a}}_{k}^{IMU}$ denote the angular velocity and linear acceleration of IMU measurements at time step *k*. The measurement models are [29]:

(17) $$\left\{\begin{array}{c} {\tilde{\omega }}_{k}^{IMU}=\omega_{k}+b_{k}^{g}+\eta^{g}\\ {\tilde{a}}_{k}^{IMU}=R_{b_{k}}^{w}\left(a_{k}-g^{w}\right)+b_{k}^{a}+\eta^{a} \end{array}\right.$$

where $\omega_{k}$ and $a_{k}$ are the true angular velocity and linear acceleration at time step *k*, $b_{k}^{g}$ and $b_{k}^{a}$ are the gyroscope and accelerometer biases at time step *k*, $\eta^{g}$ and $\eta^{a}$ are white Gaussian noise, $g^{w}$ is the gravity vector in the world frame, and $R_{b_{k}}^{w}$ represents the rotation matrix from the robot body frame to the world frame at time step *k*.

The continuous-time kinematics of the robot are given by [30]:

(18) $$\left\{\begin{array}{c} {\dot{R}}_{b_{t}}^{w}=R_{b_{t}}^{w}{\left[\omega_{t}\right]}_{\times }\\ {\dot{v}}_{t}^{w}=R_{b_{t}}^{w}a_{t}+g^{w}\\ {\dot{p}}_{t}^{w}=v_{t}^{w} \end{array}\right.$$

where ${\left[\omega_{t}\right]}_{\times }$ denotes the skew-symmetric matrix of the angular velocity vector at time *t*, $\omega_{t}$ and $a_{t}$ are the true angular velocity and linear acceleration at time *t*, $R_{b_{t}}^{w}$, $v_{t}^{w}$, and $p_{t}^{w}$ are the rotation, velocity, and position in the world frame at time *t*, respectively.

#### 2.3.2. IMU Preintegration Definition

Direct integration of IMU measurements between keyframes *i* and *j* depends on the initial state $R_{b_{i}}^{w}$, $v_{i}^{w}$, $p_{i}^{w}$, requiring re-integration whenever these states change, which leads to high computational cost [24]. To avoid this, we adopt the preintegration technique, which expresses relative motion increments in the local frame of keyframe *i*.

Define the IMU preintegrated measurements $z_{ij}^{IMU}$ between keyframes *i* and *j* as:

(19) $$\left\{\begin{array}{c} \Delta {\tilde{R}}_{ij}^{IMU}=\prod_{k=i}^{j-1}exp(\left({\tilde{\omega }}_{k}^{IMU}-b_{k}^{g}\right)\Delta t)\\ \Delta {\tilde{v}}_{ij}^{IMU}=\overset{j-1}{\underset{k=i}{\Sigma }}\Delta {\tilde{R}}_{ik}^{IMU}\left({\tilde{a}}_{k}^{IMU}-b_{k}^{a}\right)\Delta t\\ \Delta {\tilde{p}}_{ij}^{IMU}=\overset{j-1}{\underset{k=i}{\Sigma }}\left[\Delta {\tilde{v}}_{ik}^{IMU}\Delta t+\frac{1}{2}\Delta {\tilde{R}}_{ik}^{IMU}\left({\tilde{a}}_{k}^{IMU}-b_{k}^{a}\right)\Delta t^{2}\right] \end{array}\right.$$

with initial conditions $\Delta {\tilde{R}}_{ii}^{IMU}=I$, $\Delta {\tilde{v}}_{ii}^{IMU}=0$, $\Delta {\tilde{p}}_{ii}^{IMU}=0$, where $\Delta {\tilde{R}}_{ij}^{IMU}$, $\Delta {\tilde{v}}_{ij}^{IMU}$, and $\Delta {\tilde{p}}_{ij}^{IMU}$ are the preintegrated rotation, velocity, and position measurements from keyframe *i* to *j*, Δt is the sampling time interval, $\Delta {\tilde{R}}_{ik}^{IMU}$, $\Delta {\tilde{v}}_{ik}^{IMU}$, and $\Delta {\tilde{p}}_{ik}^{IMU}$ are the preintegrated rotation, velocity, and position increments from keyframe *i* to *k*, and exp(·) is the exponential mapping from the Lie algebra to the Lie group.

In practice, we use the midpoint integration method for better accuracy:

(20) $$\left\{\begin{array}{c} \Delta {\tilde{R}}_{k+1}^{IMU}=\Delta {\tilde{R}}_{k}^{IMU}exp\left(\frac{\left({\tilde{\omega }}_{k}^{IMU}-b_{k}^{g}\right)+\left({\tilde{\omega }}_{k+1}^{IMU}-b_{k+1}^{g}\right)}{2}\Delta t\right)\\ \Delta {\tilde{v}}_{k+1}^{IMU}=\Delta {\tilde{v}}_{k}^{IMU}+\Delta {\tilde{R}}_{k}^{IMU}\frac{\left({\tilde{a}}_{k}^{IMU}-b_{k}^{a}\right)+\left({\tilde{a}}_{k+1}^{IMU}-b_{k+1}^{a}\right)}{2}\Delta t\\ \Delta {\tilde{p}}_{k+1}^{IMU}=\Delta {\tilde{p}}_{k}^{IMU}+\Delta {\tilde{v}}_{k}^{IMU}\Delta t+\frac{1}{2}\Delta {\tilde{R}}_{k}^{IMU}\frac{\left({\tilde{a}}_{k}^{IMU}-b_{k}^{a}\right)+\left({\tilde{a}}_{k+1}^{IMU}-b_{k+1}^{a}\right)}{2}\Delta t^{2} \end{array}\right.$$

#### 2.3.3. Uncertainty Propagation and Jacobians

The uncertainty of the preintegrated measurements is propagated through an error-state Kalman filter. The linearized error-state dynamics are given by:

(21) $$\delta z_{k+1}^{IMU}=F_{k}\delta z_{k}^{IMU}+G_{k}n_{k}$$

where $\delta z_{k}^{IMU}$ and $n_{k}$ are the error-state vector and measurement noise vector of IMU at time step *k*, and $F_{k}$ and $G_{k}$ are the Jacobian matrices derived from the motion model at time step *k*.

The IMU error-state covariance is propagated as follows:

(22) $$P_{k+1}^{IMU}=F_{k}P_{k}^{IMU}F_{k}^{T}+G_{k}Q^{IMU}G_{k}^{T}$$

starting from $P_{i}^{IMU}=0$, where $Q^{IMU}$ is the IMU noise covariance matrix, and $P_{k}^{IMU}$ is the IMU error-state covariance matrix at time step *k*. The final covariance $\Sigma_{ij}^{IMU}$ is the 9×9 submatrix of $P_{j}^{IMU}$ corresponding to rotation error δθ, velocity error δv, and position error δp.

The IMU information matrix used in optimization is the inverse of the covariance:

(23) $$\Omega_{IMU}={\left(\Sigma_{ij}^{IMU}\right)}^{-1}$$

The Jacobians of the preintegrated measurements with respect to bias changes are approximated by first-order expansion:

(24) $$\left\{\begin{array}{c} \Delta {\tilde{R}}_{ij}^{IMU}\left(b_{i}^{g}\right)\approx \Delta {\tilde{R}}_{ij}^{IMU}\exp\left(\frac{\partial \Delta {\tilde{R}}_{ij}^{IMU}}{\partial b^{g}}\delta b_{i}^{g}\right)\\ \Delta {\tilde{v}}_{ij}^{IMU}\left(b_{i}^{g},b_{i}^{a}\right)\approx \Delta {\tilde{v}}_{ij}^{IMU}+\frac{\partial \Delta {\tilde{v}}_{ij}^{IMU}}{\partial b^{g}}\delta b_{i}^{g}+\frac{\partial \Delta {\tilde{v}}_{ij}^{IMU}}{\partial b^{a}}\delta b_{i}^{a}\\ \Delta {\tilde{p}}_{ij}^{IMU}\left(b_{i}^{g},b_{i}^{a}\right)\approx \Delta {\tilde{p}}_{ij}^{IMU}+\frac{\partial \Delta {\tilde{p}}_{ij}^{IMU}}{\partial b^{g}}\delta b_{i}^{g}+\frac{\partial \Delta {\tilde{p}}_{ij}^{IMU}}{\partial b^{a}}\delta b_{i}^{a} \end{array}\right.$$

where $b_{i}^{g}$ and $b_{i}^{a}$ are the gyroscope and accelerometer biases at keyframes *i*; $\delta b_{i}^{g}$ and $\delta b_{i}^{a}$ are their corresponding errors. These Jacobians are computed recursively during preintegration and allow efficient bias updates without re-integration.

#### 2.3.4. IMU Preintegration Residual

The IMU preintegration residual is then defined as:

(25) $$r_{IMU}\left(z_{ij}^{IMU},X\right)=\left[\begin{array}{c} Log({\left(\Delta {\tilde{R}}_{ij}^{IMU}\left(b^{g}\right)\right)}^{T}{\left(R_{i}^{w}\right)}^{T}R_{j}^{w})\\ {\left(R_{i}^{w}\right)}^{T}\left(v_{j}^{w}-v_{i}^{w}-g^{w}\Delta t_{ij}\right)-\Delta {\tilde{v}}_{ij}^{IMU}\left(b^{g},b^{a}\right)\\ {\left(R_{i}^{w}\right)}^{T}\left(p_{j}^{w}-p_{i}^{w}-v_{i}^{w}\Delta t_{ij}-\frac{1}{2}g^{w}\Delta t_{ij}^{2}\right)-\Delta {\tilde{p}}_{ij}^{IMU}\left(b^{g},b^{a}\right) \end{array}\right]$$

where log(·) maps a rotation matrix to its corresponding rotation vector.

The IMU preintegration technique decouples the relative motion from the absolute initial state, allowing efficient bias updates and avoiding repeated integration. The preintegrated measurements ${\tilde{R}}_{b_{i}}^{w}$, ${\tilde{v}}_{i}^{w}$, ${\tilde{p}}_{i}^{w}$, along with their covariance $\Sigma_{ij}^{IMU}$ and Jacobians with respect to biases, provide a compact and efficient representation for IMU constraints in the factor graph optimization framework.

### 2.4. Wheel Odometry Preintegration

#### 2.4.1. Wheel Odometry Measurement Model

Wheel odometry provides incremental displacement measurements in the robot body frame. Let $\Delta {\tilde{x}}_{k}^{b}$, $\Delta {\tilde{y}}_{k}^{b}$, $\Delta {\tilde{\theta }}_{k}^{b}$ denote the raw wheel odometry measurements at time step *k*. The measurement model is:

(26) $$\left\{\begin{array}{c} \Delta {\tilde{x}}_{k}^{b}=\Delta x_{k}^{b}+\eta_{x}\\ \Delta {\tilde{y}}_{k}^{b}=0+\eta_{y}\\ \Delta {\tilde{\theta }}_{k}^{b}=\Delta \theta_{k}^{b}+\eta_{\theta } \end{array}\right.$$

where $\Delta x_{k}^{b}$ and $\Delta \theta_{k}^{b}$ are the true displacement and heading change at time step *k*, and $\eta_{x}$, $\eta_{y}$, $\eta_{\theta }$ are zero-mean Gaussian noise. The assumption $\Delta {\tilde{y}}_{k}^{b}\approx 0$ holds for planar motion on flat ground.

#### 2.4.2. Wheel Odometry Preintegration Definition

To avoid re-integration due to state changes and the associated computational cost, similar to IMU preintegration, we define the wheel odometry preintegrated measurements between keyframes *i* and *j* as:

(27) $$\left\{\begin{array}{c} \Delta {\tilde{x}}_{ij}^{b}=\sum_{k=i}^{j-1}\Delta {\tilde{x}}_{k}^{b}\\ \Delta {\tilde{y}}_{ij}^{b}=\sum_{k=i}^{j-1}\Delta {\tilde{y}}_{k}^{b}\\ \Delta {\tilde{\theta }}_{ij}^{b}=\sum_{k=i}^{j-1}\Delta {\tilde{\theta }}_{k}^{b} \end{array}\right.$$

where $\Delta {\tilde{x}}_{ij}^{b}$ and $\Delta {\tilde{y}}_{ij}^{b}$ denote the preintegrated forward and lateral displacements, respectively, and $\Delta {\tilde{\theta }}_{ij}^{b}$ denotes the preintegrated heading change, all expressed in the robot body frame $\left\{b\right\}$.

For notational convenience, we group them into a vector:

(28) $$\Delta {\tilde{p}}_{ij}^{b}=\left[\begin{array}{c} \Delta {\tilde{x}}_{ij}^{b}\\ \Delta {\tilde{y}}_{ij}^{b}\\ \Delta {\tilde{\theta }}_{ij}^{b} \end{array}\right]$$

where $\Delta {\tilde{p}}_{ij}^{b}$ is the preintegrated relative position measurement from keyframe *i* to *j* in the robot body frame.

#### 2.4.3. Uncertainty Propagation for Wheel Odometry Preintegration

The uncertainty of the wheel odometry preintegrated measurements is propagated similarly to IMU. Define the wheel odometry error-state vector at time step *k*:

(29) $$\delta z_{k}^{WO}={\left[\begin{array}{ccc} \delta \Delta x_{k}^{b} & \delta \Delta y_{k}^{b} & \delta \Delta \theta_{k}^{b} \end{array}\right]}^{T}$$

where $\delta \Delta x_{k}^{b}$, $\delta \Delta y_{k}^{b}$, and $\delta \Delta \theta_{k}^{b}$ are the errors of the raw wheel odometry measurements at time step *k*.

The wheel odometry error-state propagation is given by:

(30) $$\delta z_{k+1}^{WO}=F_{k}^{WO}\delta z_{k}^{WO}+G_{k}^{WO}n_{k}^{WO}$$

where $F_{k}^{WO}$ and $G_{k}^{WO}$ are the state transition and noise input Jacobian matrices at time step *k*, and $n_{k}^{WO}$ is the measurement noise vector at time step *k*.

The covariance matrix $\Sigma_{ij}^{WO}$ is propagated by iteratively applying:

(31) $$P_{k+1}^{\mathrm{WO}}=F_{k}^{\mathrm{WO}}P_{k}^{\mathrm{WO}}{\left(F_{k}^{\mathrm{WO}}\right)}^{T}+G_{k}^{\mathrm{WO}}Q^{\mathrm{WO}}{\left(G_{k}^{\mathrm{WO}}\right)}^{T}$$

starting from $P_{i}^{WO}=0$ (the covariance matrix of the wheel odometer error state at keyframes *i*), where $Q^{WO}$ is the noise covariance matrix of the wheel odometry.

The wheel odometry information matrix used in optimization is the inverse of the covariance:

(32) $$\Omega_{WO}={\left(\Sigma_{ij}^{WO}\right)}^{-1}$$

#### 2.4.4. Wheel Odometry Preintegration Residual

The wheel odometry preintegration residual is then defined as:

(33) $$r_{WO}\left(z_{ij}^{WO},X\right)=\left[\begin{array}{c} \Delta {\tilde{x}}_{ij}^{b}-\left(\cos\left(\theta_{i}\right)\left(x_{j}^{w}-x_{i}^{w}\right)+\sin\left(\theta_{i}\right)\left(y_{j}^{w}-y_{i}^{w}\right)\right)\\ \Delta {\tilde{y}}_{ij}^{b}-\left(-\sin\left(\theta_{i}\right)\left(x_{j}^{w}-x_{i}^{w}\right)+\cos\left(\theta_{i}\right)\left(y_{j}^{w}-y_{i}^{w}\right)\right)\\ \Delta {\tilde{\theta }}_{ij}^{b}-\left(\theta_{j}^{w}-\theta_{i}^{w}\right) \end{array}\right]$$

where $\left(x_{i}^{w},y_{i}^{w},\theta_{i}^{w}\right)$ and $\left(x_{j}^{w},y_{j}^{w},\theta_{j}^{w}\right)$ are the robot poses in the world frame at keyframes *i* and *j*, with $cos\;\theta_{i}$ and $sin\;\theta_{i}$ being the elements of the rotation matrix that transforms the global displacement into the body frame.

This residual constraint ensures the consistency of the robot’s planar motion. By leveraging the drift-free nature of the wheeled odometer, it effectively suppresses horizontal integration drift in IMU integration and provides an absolute metric scale for the monocular vision system.

### 2.5. Tightly Coupled Nonlinear Optimization

The estimated state vector X includes the position, orientation, velocity, and IMU biases for a series of keyframes. The tightly coupled global objective function is:

(34) $$J\left(X\right)=\sum_{VO}\rho (r_{VO}^{T}\Omega_{VO}r_{VO})+\sum_{Tag}\rho (r_{Tag}^{T}\Omega_{Tag}r_{Tag})+\sum_{IMU}\rho (r_{IMU}^{T}\Omega_{IMU}r_{IMU})+\sum_{WO}\rho (r_{WO}^{T}\Omega_{WO}r_{WO})$$

where ρ(·) is the Huber robust kernel applied to the squared Mahalanobis distance $e=\sqrt{r^{T}\Omega r}$, defined as:

(35) $$\rho \left(e\right)=\left\{\begin{array}{cc} \frac{1}{2}e^{2}, & \left|e\right|\leq \delta \\ \delta (\left|e\right|-\frac{1}{2}\delta ), & \left|e\right|>\delta \end{array}\right.$$

and δ is a tuning parameter (set to 1.0 in our experiments).

**Figure 4:**
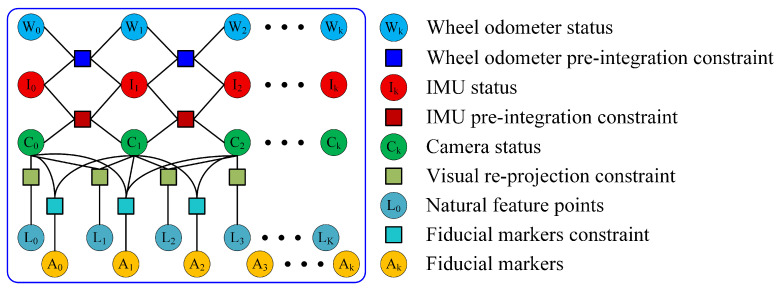
Factor Graph Optimization Framework.

The optimal state $X^{*}$ is obtained by minimizing J(X) using the Gauss–Newton method. All observations are tightly fused within a factor graph optimization framework, as illustrated in Figure 4. In this factor graph, circular nodes denote the state variables to be estimated, and square factors represent the various observational constraints. Edges connect state nodes to observed factors, with their strengths determined by the corresponding uncertainty models. Through this optimization framework, the system outputs high-precision robot poses in real time. The proposed framework achieves real-time performance with the following computational complexity: sensor preintegration O(N), where *N* is the number of sensor measurements between keyframes; visual processing $O(N_{pix}+N_{feat}logN_{feat})$, where $N_{pix}$ is the number of image pixels and $N_{feat}$ is the number of Oriented FAST and Rotated BRIEF (ORB) features per frame; and factor graph optimization $O(S^{3})$ with bounded *S* (constant per iteration), where *S* is the dimension of the sliding window state vector.

### 2.6. Experimental Setup

#### 2.6.1. Experimental Site and Equipment

Experiments were conducted in a commercial caged chicken house in Raoping County, Guangdong Province, China. The house features a typical stacked cage structure containing multiple corridors, each approximately 80 m long and 1 m wide, with metal cages on both sides and a concrete floor. A manure pit is located at the end of each corridor.

**Figure 5:**
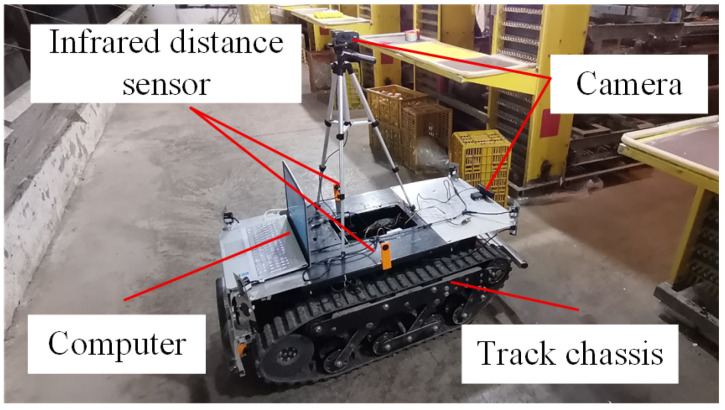
Experimental platform and sensor configuration.

The experimental platform was built on a Guoxing Intelligent Safari-600T mini tracked chassis. It was equipped with two cameras (C925E, Logitech, Newark, CA, USA), an IMU (A100, Glonavin, Changsha, China), two wheel encoders (BS38H, Bode, Wuxi, China), a microcontroller (STM32F103ZET6, Alientek, Guangzhou, China), and two infrared distance sensors (WT53R, Wit Motion, Shenzhen, China). The upper-level algorithms ran on an HP RMN TPN-C122 computer (Intel Core i7-5500U, 4 GB RAM; HP, Palo Alto, CA, USA). The camera was processed at 10 Hz to balance computational load and real-time performance, which is justified by the robot’s operating speed. The camera was pre-calibrated and time-synchronized with the IMU and wheel encoder at the system level. The sensor configuration of the experimental platform is illustrated in Figure 5.

#### 2.6.2. Data Acquisition

According to the operational requirements of poultry monitoring systems, the optimal inspection speed is 0.1–0.2 m/s [31,32]. Therefore, two speeds were selected: 0.116 m/s and 0.232 m/s, covering the typical speed range. The robot traveled from the corridor entrance to the end and back (one round trip). Each speed was repeated 3 times to eliminate random errors.

**Figure 6:**
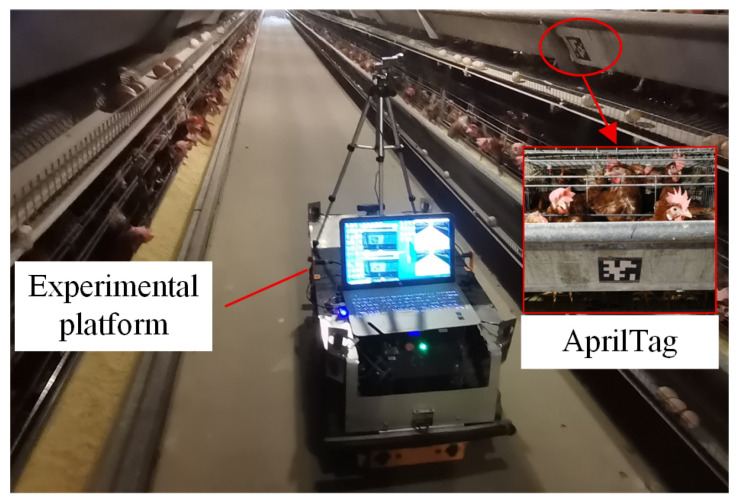
Data acquisition process.

To provide global pose constraints, AprilTags (Tag36h11, 10 cm side length) were attached to the feeder surface at 10 m intervals along the corridor (8 tags in total). The depth axis of each tag corresponds to the corridor width direction, and its lateral axis corresponds to the corridor length direction. The tag plane was kept perpendicular to the ground to ensure detection stability. The ground truth trajectory was obtained by fusing two complementary measurements. Two high-precision infrared distance sensors mounted on the left and right sides of the robot measured the distances to the feeder wall, from which the robot’s lateral position (x-coordinate) was computed via triangulation. The longitudinal position (y-coordinate) was obtained through the detection of AprilTag: when the robot passed by on its side, these tags provided an absolute reference. Fusing these two sources yielded the full planar pose of the robot as the ground truth. Data were collected using ROS Noetic (Ubuntu 20.04) and processed with Python 3.8.12. The data acquisition process is illustrated in Figure 6.

#### 2.6.3. Evaluation Metrics

The following metrics were used to evaluate the performance of the proposed CVIWM method. The lateral deviation is defined as the perpendicular distance from the robot to the corridor centerline, which directly reflects the robot’s centering accuracy and is critical for avoiding collisions with cages in the narrow corridor. The longitudinal deviation is defined as the distance error along the corridor direction, which reflects the absolute positioning accuracy and is essential for ensuring the robot stops precisely at the designated cages for reliable image acquisition. The root mean square error (RMSE) of the lateral and longitudinal deviations is also computed to provide a comprehensive measure of positioning accuracy.

## 3. Results

### 3.1. Fiducial Marker Positioning Accuracy

**Figure 7:**
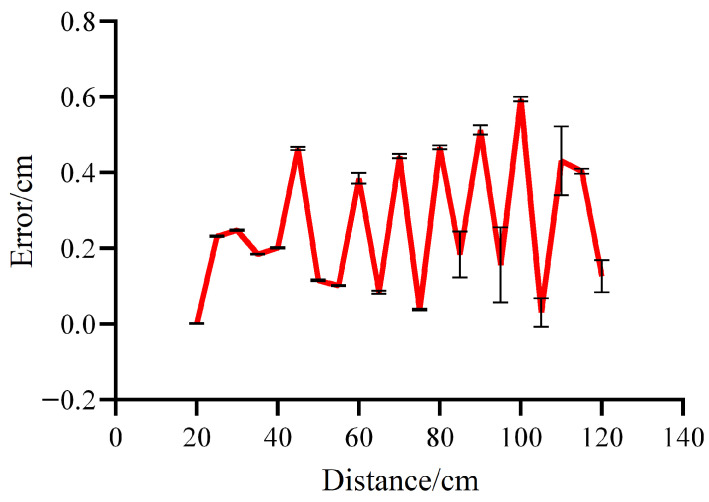
Positioning accuracy of the fiducial marker in the depth direction.

To verify the reliability of fiducial markers as absolute pose constraints, positioning accuracy was evaluated in the depth direction (corridor width direction, distance 0.2–1.2 m). Results are shown in Figure 7. The average positioning error was 0.257 cm, with a standard deviation of 0.179 cm and RMSE of 0.313 cm. The maximum effective recognition distance reached 6.6 m. Positioning accuracy in the lateral direction (corridor length direction) was comparable, with an average error <0.31 cm. These results demonstrate that fiducial markers provide sub-centimeter-level, high-confidence absolute pose constraints for the CVIWM method.

### 3.2. State Estimation Performance in Structurally Degraded Environments

#### 3.2.1. Qualitative Trajectory Analysis

**Figure 8:**
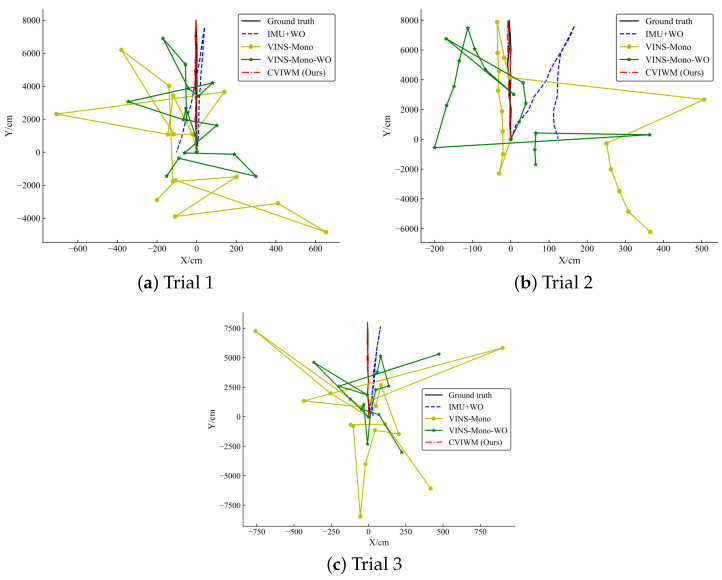
Trajectory comparison of different positioning methods at 0.116 m/s.

**Figure 9:**
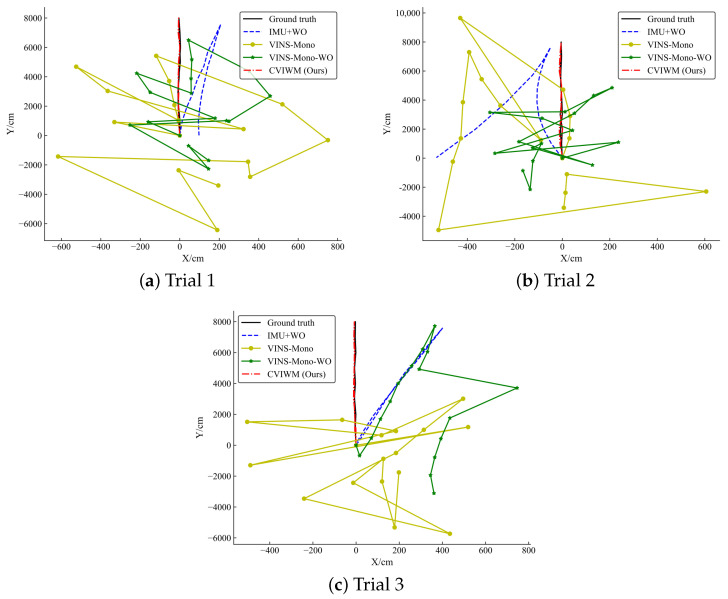
Trajectory comparison of different positioning methods at 0.232 m/s.

All methods were evaluated on the identical dataset collected from the experimental corridor. Figure 8 (0.116 m/s) and Figure 9 (0.232 m/s) present the trajectory comparisons between the proposed CVIWM and the three baseline methods: inertial navigation with wheel odometry fusion [33], VINS-Mono [24], and VINS-Mono-WO [34]. Overall, as the travel speed increases, the deviation between the estimated trajectories and the ground truth tends to grow for all compared methods. In contrast, CVIWM maintains good trajectory alignment with the true path at both speeds.

The trajectory of the inertial navigation method exhibits clear error accumulation characteristics. This phenomenon primarily stems from the inherent limitations of inertial navigation systems, where heading angle errors accumulate over time. In long straight corridors, even a small heading angle deviation is progressively amplified by integration, ultimately causing severe lateral and longitudinal deviation from the true path. Since the test path involved a round trip, the forward and backward runs had opposite error directions. When switching direction at the end of the corridor, some lateral errors canceled each other out; therefore, the error growth during the round trip was mitigated compared to that of a one-way run.

The trajectory of the VINS-Mono method shows severe drift and non-physical jumps (e.g., the longitudinal coordinate jumps from ∼40 m to ∼−30 m). This is attributed to the sparse and repetitive textures on both sides of the caged chicken house corridor, which cause ambiguity in visual feature matching. As a result, the system cannot obtain reliable visual motion estimates and degenerates into pure inertial integration, causing a sharp increase in drift. By incorporating wheel odometry, the VINS-Mono-WO method improves drift to some extent, but its trajectory still deviates considerably from the true path. Moreover, the positioning accuracy of VINS-Mono-WO is even lower than that of the simpler inertial navigation system. This finding indicates that in environments with degraded structural features, poorly qualified visual measurements, if not effectively screened and corrected, introduce errors that far outweigh the benefits they provide. Both of these methods produce trajectories that have completely deviated from the true path, demonstrating that in structurally degraded environments such as caged chicken houses, feature-matching-based localization methods are highly prone to failure when the environment lacks rich and stable features.

By contrast, CVIWM overcomes the above limitations by incorporating the drift-free planar constraints of wheel odometry and absolute pose corrections from fiducial markers. Its estimated trajectory closely follows the actual robot path, with both lateral and longitudinal deviations remaining within small bounds, validating the effectiveness and robustness of the method in structurally degraded environments.

#### 3.2.2. Quantitative Error Analysis

Quantitative results are presented in Table 1 (0.116 m/s) and Table 2 (0.232 m/s). As speed increased, positioning errors of all methods increased, with CVIWM showing the smallest degradation. All values are averaged over three repeated trials.

For the inertial navigation method, the maximum mean lateral and longitudinal deviations reached 155.214 cm and 178.798 cm, respectively (at 0.232 m/s). Such substantial errors far exceed the accuracy requirements for caged chicken house inspection. The VINS-Mono method exhibited even larger positioning errors, with maximum mean lateral and longitudinal deviations of 253.939 cm and 3782.568 cm (at 0.232 m/s), accompanied by significantly high standard deviations, indicating severe fluctuations and a lack of consistency. This effectively renders it unusable in this environment. Notably, the longitudinal deviation far exceeded the lateral deviation, further confirming the trajectory analysis conclusion: in environments with sparse and repetitive textures, visual feature matching fails, causing the system to degrade into pure inertial integration, where longitudinal errors grow rapidly. The VINS-Mono-WO method showed reduced errors (maximum mean lateral deviation of 195.106 cm and maximum mean longitudinal deviation of 1991.345 cm at 0.232 m/s) compared to VINS-Mono, but its positioning accuracy remained far inferior to that of the much simpler inertial navigation system, falling well short of practical requirements. This result further corroborates the earlier conclusion: in structurally degraded environments, poor-quality visual observations introduce errors that outweigh any corrective benefits they bring to the fusion system.

**Figure 10:**
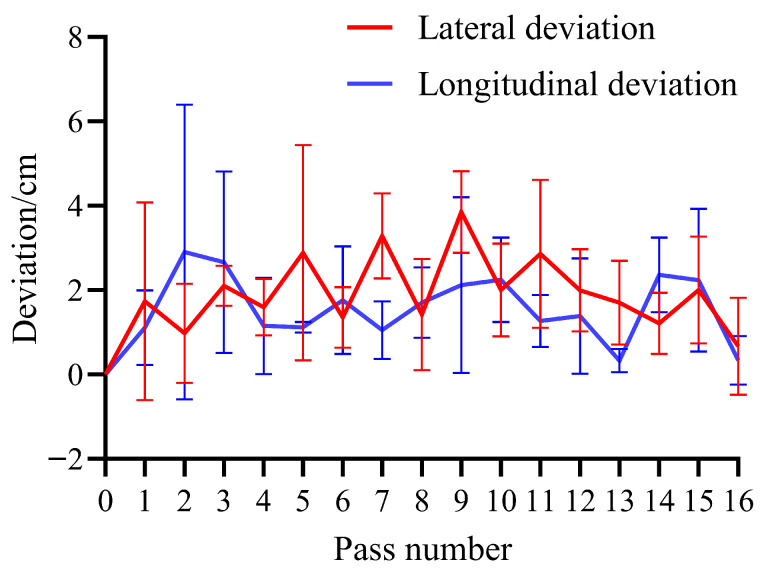
Deviation at the speed of 0.116 m/s.

**Figure 11:**
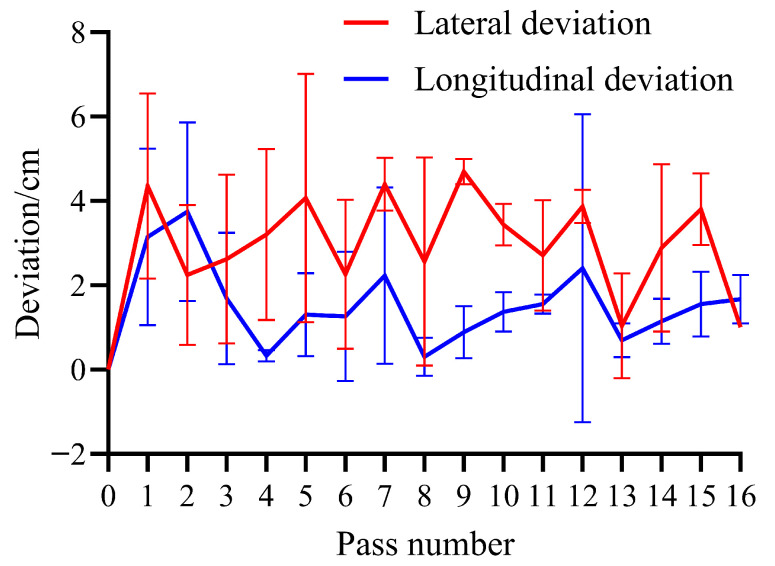
Deviation at the speed of 0.232 m/s.

The proposed CVIWM method significantly outperformed the comparison methods in terms of positioning accuracy. At 0.116 m/s, the mean lateral and longitudinal deviations were 1.863 cm and 1.516 cm, with standard deviations of 1.403 cm and 1.406 cm, respectively. At 0.232 m/s, the mean lateral and longitudinal deviations were 2.893 cm and 1.487 cm, with standard deviations of 1.814 cm and 1.506 cm, respectively. Based on these mean deviations, the overall mean positioning error at 0.116 m/s and 0.232 m/s was approximately 2.402 cm and 3.253 cm, respectively, calculated as the root mean square of the mean lateral and mean longitudinal deviations. The positioning deviations recorded each time the robot passed a sampling point are shown in Figure 10 and Figure 11. As the traveling speed increases, the lateral deviation shows a marked increase, while the longitudinal deviation remains relatively stable. This is because heading errors accumulate rapidly at higher speeds, exacerbating lateral drift. In contrast, the longitudinal deviation is primarily governed by the wheel odometry scale factor, resulting in relatively linear error growth, and periodic corrections from the fiducial markers effectively suppress longitudinal drift.

In terms of RMSE, CVIWM achieves lateral/longitudinal values of 2.332/2.068 cm at 0.116 m/s and 3.414/2.116 cm at 0.232 m/s, demonstrating centimeter-level accuracy and excellent stability. The lateral RMSE increases by 46% (from 2.332 cm to 3.414 cm) when the speed doubles, which is expected as higher speeds amplify residual heading errors. Importantly, even at the higher speed of 0.232 m/s (the upper limit of the practical operating range for poultry inspection [31,32]), the positioning error remains within the centimeter level. Given a typical cage width of 60–70 cm and the camera’s horizontal field of view, this accuracy ensures that the target cage stays within the central field of view, enabling reliable health monitoring. Regarding computational efficiency, the proposed CVIWM system achieved an average processing latency of 88 ms per frame (≈11 Hz), which exceeds the 10 Hz camera input rate, confirming its real-time capability for poultry house inspection tasks.

### 3.3. Impact of Positioning Accuracy on Poultry Health Monitoring

#### 3.3.1. Effect on Image Acquisition

Based on the camera’s parameters (70.5 cm field of view at 50 cm distance, 1280×720 resolution), the relationship between lateral deviation and pixel shift is approximately linear: a 3 cm deviation results in a 55-pixel shift, a 5 cm deviation results in a 91-pixel shift, and a 10 cm deviation results in a 182-pixel shift. When the lateral deviation exceeds 10 cm, the corresponding pixel shift surpasses 180 pixels, which is likely to cause the target cage to deviate significantly from the image center, thereby compromising the accuracy of subsequent health detection algorithms.

#### 3.3.2. Actual Detection Performance of CVIWM

After the robot stopped at the designated cages guided by the CVIWM-estimated poses, images were captured for health monitoring. Using CVIWM, the average stopping position errors were 2.8 cm (0.116 m/s) and 3.5 cm (0.232 m/s), with a maximum error of <4.5 cm. This high precision corresponds to an image shift of <83 pixels, ensuring the target cage remains well-centered.

**Figure 12:**
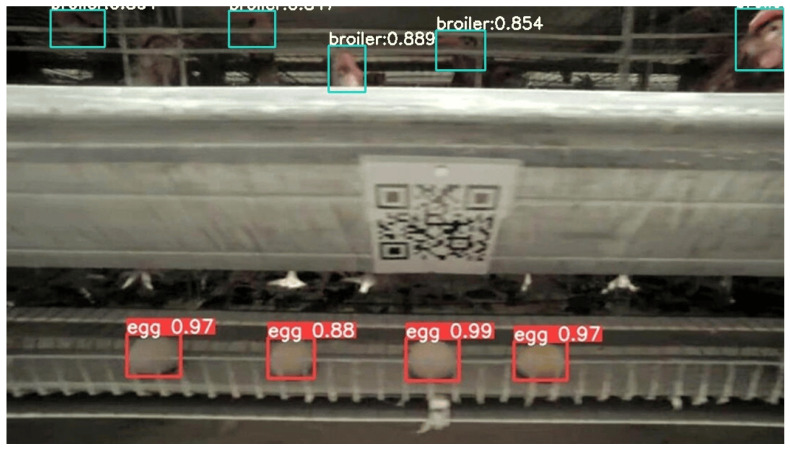
Examples of poultry health monitoring results using images acquired by the CVIWM-guided robot.

It is important to clarify that the poultry detection models used in this study are pre-trained and fixed [35,36], and CVIWM does not improve their inherent detection accuracy. Notably, the training data for these models were collected using the same robotic platform, where CVIWM ensured accurate cage-level positioning during data acquisition [35,36]. The key contribution of CVIWM is to guarantee that the robot stops at the correct position, enabling the camera to capture images from the intended cage. Without this centimeter-level positioning, the captured images would be misaligned, rendering even the best detection models ineffective. Under this setup, the pre-trained models achieved a dead hen detection accuracy of 95.7% and an egg detection accuracy of 98.9% across all 60 cages and 26 dead hen samples (see Figure 12). These results demonstrate that CVIWM provides the essential positioning foundation for automated poultry health monitoring.

## 4. Discussion

### 4.1. Comparison with Existing Methods

Compared with existing methods, CVIWM demonstrates clear advantages in both accuracy and practicality (Table 3). For instance, while RFID [37] and UWB [38] offer accuracies of 6 cm and 17 cm, respectively, they suffer from short reading ranges or electromagnetic interference [39]. Vision-based methods, such as Zhang et al.’s [7] approach, rely on densely distributed markers (1–5 m spacing) to achieve accuracies of 5.6–7.6 cm. The visual-marker-inertial fusion system using sliding window optimization integrates AprilTag observations to provide absolute pose corrections [25], effectively reducing drift in weakly textured environments. However, without wheel odometry constraints, it remains susceptible to residual drift between markers, particularly in long straight corridors.

The LIO-SAM+AprilTag method [6] can achieve relatively high accuracy (4.1–7.0 cm). However, its performance largely depends on the geometric features extracted from the LiDAR point cloud. In geometrically degraded environments, such as the repetitive corridors of a caged chicken house, relying solely on LiDAR and IMU still makes the system prone to drift. Even with the assistance of AprilTags, point cloud degeneracy can lead to significant lateral drift. Furthermore, the high cost of LiDAR sensors limits its feasibility for large-scale deployment.

In contrast, CVIWM integrates vision, IMU, wheel odometry, and AprilTags into a factor graph optimization framework. By tightly coupling these complementary sensors, it uses wheel odometry to suppress IMU drift and vision-based markers to provide absolute corrections, achieving high accuracy (2.4–3.3 cm). Moreover, it requires only sparse AprilTags (10 m spacing) and low-cost vision sensors. Even in weak-texture regions between tags (e.g., chicken coop grids), wheel odometry maintains short-term precision, offering a practical, cost-effective solution for large poultry houses.

### 4.2. Method Advantages and Innovations

CVIWM has three main advantages: (1) wheel odometry preintegration provides drift-free planar constraints, suppressing IMU horizontal drift and providing absolute scale; (2) fiducial markers provide periodic absolute pose corrections, resetting accumulated errors; (3) structural degradation adaptability enables stable positioning in long straight corridors with sparse textures.

### 4.3. Engineering Value for Smart Farming

The engineering value of CVIWM is threefold. First, by ensuring target cages remain in the central region of captured images (image shift <83 pixels), it directly supports downstream poultry health monitoring tasks, achieving high detection accuracy (as detailed in Section 3.3.2). Second, it offers significant economic and operational advantages, including low-cost visual sensors (instead of LiDAR) and easy deployment with sparse markers (10 m spacing) and no pre-built map requirement. Third, it demonstrates high reliability, with no significant drift over 80 m of continuous operation. These combined attributes make CVIWM a practical and scalable solution for automated inspection in commercial poultry houses.

### 4.4. Limitations and Future Work

While CVIWM demonstrated high positioning accuracy over an 80 m corridor, several limitations remain to be addressed. First, although the current experiments accounted for some dust and lighting variations, different lighting may still degrade performance. Thus, further investigation under more challenging conditions—wet floors, wheel slippage, heavy dust, poor lighting, occluded or damaged AprilTags, varying tag densities, and complex motions—is needed to evaluate system robustness, motivating future work on slip compensation, online calibration [40], and multi-modal sensing using IR-reflective or thermal markers [41,42]. Second, dynamic obstacles were not considered. Integrating dynamic obstacle detection and tracking would be necessary for safe operation in human–robot shared environments [43]. Third, although several hours of experiments were conducted in commercial poultry houses, the long-term deployment feasibility and reliability of the system require further validation through extended field testing. In addition, the energy consumption of the overall sensor fusion and navigation framework needs to be further evaluated to achieve optimal power management.

## 5. Conclusions

This paper proposed CVIWM, a tightly coupled state estimation method for poultry house inspection robots operating in structurally degraded environments. The method integrates visual, IMU, wheel odometry, and fiducial marker observations within a factor graph optimization framework. Specifically, wheel odometry preintegration provides drift-free planar constraints to suppress IMU horizontal drift and provide an absolute scale for monocular vision, while sparse fiducial markers (AprilTags) serve as absolute pose anchors to periodically reset accumulated errors. Experimental results in an 80 m long poultry house corridor demonstrated that CVIWM achieves centimeter-level positioning accuracy, with average positioning errors of 2.402 cm at 0.116 m/s and 3.253 cm at 0.232 m/s. This high-precision positioning ensured reliable image acquisition (image shift <83 pixels), enabling subsequent poultry detection models to achieve 95.7% dead hen detection accuracy and 98.9% egg detection accuracy. By providing a low-cost, easy-to-deploy, and high-accuracy state estimation solution, CVIWM serves as a key technological enabler for automated inspection and smart livestock farming.

## Figures and Tables

**Table 1:** Positioning deviation statistics at 0.116 m/s.

Method	Mean Lateral Deviation (cm)	Lateral Deviation Std (cm)	Mean Longitudinal Deviation (cm)	Longitudinal Deviation Std (cm)
IMU+WO	60.049	31.373	169.724	123.234
VINS-Mono	184.634	206.940	3724.088	2570.585
VINS-Mono-WO	106.807	104.005	1913.330	1233.156
CVIWM (Ours)	1.863	1.403	1.516	1.406

**Table 2:** Positioning deviation statistics at 0.232 m/s.

Method	Mean Lateral Deviation (cm)	Lateral Deviation Std (cm)	Mean Longitudinal Deviation (cm)	Longitudinal Deviation Std (cm)
IMU+WO	155.214	109.714	178.798	127.704
VINS-Mono	253.939	202.898	3782.568	2564.384
VINS-Mono-WO	195.106	125.945	1991.345	1376.138
CVIWM (Ours)	2.893	1.814	1.487	1.506

**Table 3:** Performance comparison of different positioning methods.

Method	Positioning Accuracy	Remarks
RFID [37]	6 cm	Short reading range, relies on high-precision navigation
UWB [38]	17 cm	Electromagnetic interference
AprilTag + IMU + Rang [7]	5.6 cm	Marker-dependent, IMU drift
Visual + IMU + Markers [25]	—(not reported)	No wheel odometry, drift between markers
LIO-SAM + AprilTag [6]	4.1–7.0 cm	High LiDAR cost
CVIWM (Ours)	2.4–3.3 cm	Sparse markers (10 m spacing), low-cost visual sensors

## Data Availability

The data presented in this study are available on request from the corresponding author.
